# Prevention of myopia, China

**DOI:** 10.2471/BLT.19.240903

**Published:** 2020-04-28

**Authors:** Catherine Jan, Ling Li, Lisa Keay, Randall S Stafford, Nathan Congdon, Ian Morgan

**Affiliations:** a Lost Child’s Vision Project, 27 York Street, Taree, Australia 2430, Australia.; b National School of Development, Peking University, Beijing, China.; c School of Optometry and Vision Science, University of New South Wales, Sydney, Australia.; d School of Medicine, Stanford University, Stanford, United States of America.; e Centre for Public Health, Queen’s University Belfast, Belfast, Northern Ireland.; f Research School of Biology, Australian National University, Canberra, Australia.

Myopia (or short-sightedness) is a global health and social problem. Researchers have estimated that in China, myopia caused a 244 billion United States dollars loss in productivity in 2015.^1^ China’s approach to its burden of childhood myopia illustrates the problem’s magnitude, but also outlines solutions that could be potentially relevant to other countries. Here we outline China’s national myopia policy, discuss the related scientific evidence and conclude with specific recommendations for the way forward.

## Need for a national plan

Myopia control efforts have focused on children and adolescents because prevention is only possible in these age groups, progression largely occurs during this stage of life and poor vision from uncorrected myopia limits children’s educational performance. While the growing burden of myopia is a global problem, the most rapid increases and highest prevalence have been recorded in east Asian countries, including China, all sharing high-pressure school systems.^2^^,^^3^ Nationwide data from China in 2014 showed for example that an estimated 80.0% of students completing secondary education were myopic.^3^ Furthermore, increased myopia prevalence among younger children and higher rates of progression during school years have led to a growing burden (estimated at 10.0–20.0%) of high myopia (6 dioptres or more) among high school graduates. High myopia greatly increases the risk of macular atrophy, glaucoma and other causes of severe vision loss,^4^ the incidence of which is not reduced by wearing glasses.

Following a declaration in 2018 by China’s President Xi Jinping on how the high prevalence of myopia in schoolchildren affects their health and well-being, the central government’s State Council released a comprehensive plan for the prevention and control of myopia in children and adolescents.^5^ The plan describes key roles for the Ministry of Education and the National Health Commission, with support from finance and publicity departments. Essential goals, for which local governments are provided precise performance targets, include very cautious targets of reducing the myopia prevalence among schoolchildren by over 0.5% per year from 2018 to 2023, and to achieve a figure below 70.0% among high school students by 2030, through preventive interventions.^5^

## Primary prevention

Primary prevention focuses on reducing myopia incidence. The growing prevalence of high myopia and its attendant serious complications, call for primary prevention strategies, which are prioritized in the current Chinese national myopia control plan. Increasing time outdoors is most salient among these. Children living in countries and regions with a high prevalence of myopia, such as Singapore, appear to spend less time outdoors than do children in settings with low prevalence of myopia, such as Australia.^6^ This higher prevalence may be due to greater amount of time devoted to schooling and fewer venues for outdoor play in the highly urbanized areas with the greatest myopia prevalence. Time outdoors and exposure to sunlight may lead to reduced incidence of myopia owing to light-stimulated increases in dopamine release from the retina.^7^ Dopamine acts as an inhibitor of axial elongation, the anatomical basis for the development of myopia. The approach of increasing time outdoors has been validated in school-based interventional research, with a 25.0–50.0% reduction of myopia incidence observed with an additional 1–2 hours per day outdoors over the course of 1–3 years.^8^^,^^9^ This preventive strategy is most effective in younger children, typically younger than 12 years of age. A school-based national programme of two hours per day outdoors has been implemented in Taiwan, China, and a 1–2-hour daily period of outdoor time is specified in the Chinese national myopia control programme. Low cost and the potential to target China’s other childhood epidemic, obesity, make this approach very attractive, but remains only partially adopted by schools. The main barrier to implementation is the concern among parents and educators that more time outdoors will compromise educational outcomes, despite lack of evidence for such an undesirable effect.

A second opportunity for primary prevention of myopia lies in reducing the burden of schoolwork on Chinese children, particularly during the earliest educational years. The east Asian countries with highest prevalence of myopia, including China, have all implemented rigorous, highly-competitive education systems, often characterized by exposure to written homework beginning in preschool and early primary years, and extensive reliance on after-school academic activities.^2^^,^^3^ A study suggests that the positive relationship between extensive schooling and onset and progression of myopia may be mediated through work that is done at a short focal distance including reading and writing, referred to as near work.^10^ Prevention strategies built around comprehensive reductions in study time will likely not be feasible in east Asian settings, but the current plan’s call for a complete absence of written homework in the first two years of school and limitations thereafter (60 minutes for grades 3 to 6, and 90 minutes for higher grades) is an important first step.

## Secondary and tertiary prevention

With the high prevalence of childhood myopia, secondary and tertiary prevention focusing on slowing myopia progression is a government priority. Several approaches to slow the progression of childhood myopia once it has been diagnosed have recently been described.^11^ In addition to increased time outdoors, evidence suggests that the use of low-dose atropine (0.01–0.05%) eye drops,^11^ flattening of the corneal surfaces through overnight use of rigid contact lenses^11^ and use of spectacles and contact lenses designed to reduce peripheral defocus^12^ may slow myopia progression by 50.0% or more. If applied nationwide and in a timely fashion, such interventions could significantly reduce the burden of high myopia and its consequent pathology. Barriers include the potential for infection with overnight contact lens use and the fact that treatments such as low-dose atropine are not yet approved in China, therefore these interventions are not included in China’s national plan.

Access to high-quality vision assessment services is limited in China, particularly in rural areas, and the national myopia plan does not prioritize capacity-building to solve this situation. The current (thirteenth) five-year National Plan of Eye Health aims to establish at least one eye-care provider capable of determination of refractive errors in each of China’s 2800 county-level hospitals. However, since many Chinese counties have over 500 000 residents, this would still fall far short of the World Health Organization (WHO) recommendation of one refractionist per 50 000 population.

## Challenges

China’s national myopia control plan is a work in progress. The current plan promotes specific interventions, including those with a strong scientific basis,^8^ but also elements based on popular beliefs, such as adopting specific postures while reading. The government now recognizes the very different challenges presented by the separate burdens of myopia and high myopia; while conservative goals for control of the former have been set, none have yet been formulated for the latter. In addition, the need to build capacity to deliver high-quality screening, spectacle prescription and delivery in underserved rural and urban migrant settings is not fully addressed. Much research on myopia control has recently been published, but dissemination of this knowledge among Chinese senior personnel in ophthalmology, general medicine and public health remains limited.

The Chinese government’s statements on myopia and the initial release of the implementation plan have stimulated a lively debate about what works best to control myopia and what can be most effectively implemented under the China’s current circumstances. The Chinese government’s calls to increase scientific literacy in China provide a useful context for this discussion as they draw attention from various groups (such as the education and health-care sector, parents and children) on myopia and contribute to rethinking some of the current approaches to myopia control.

Since the 1960s, the backbone of the government’s strategy on myopia has been the national implementation of traditional Chinese eye exercises in schools, in theory designed to reduce myopia by stimulating the temporal meridians. Despite widespread use of these exercises in Chinese schools for decades, little scientific basis for their efficacy exists, and the prevalence of myopia has continued to increase, wasting time and resources. This approach is now being supplemented by evidence-based policies to increase time spent outdoors in schools and reduce the amount of near work. 

Remembering that measures to limit the development of myopia and high myopia are unlikely to be completely successful is important, as China will still have to provide care for people affected by myopia. Thus, the need for increased investment in primary health care, including vision care, will grow. Covering children’s glasses under China’s national medical insurance should be considered. 

## Global significance

As the proportion of children attending school continues to grow in low- and middle-income countries, other countries will need to take preventive action to avoid a growing burden of myopia. These countries could learn from China’s experience in blending proven myopia prevention measures, such as increased time outdoors and reduced schoolwork in the primary years, with an enhanced infrastructure to deliver high-quality refractive services throughout the country.
